# Accuracy and reproducibility of large language model measurements of liver metastases: comparison with radiologist measurements

**DOI:** 10.1007/s11604-025-01884-5

**Published:** 2025-10-04

**Authors:** Haruto Sugawara, Akiyo Takada, Shimpei Kato

**Affiliations:** 1 Department of Medical Imaging, The Ottawa Hospital, University of Ottawa, 501 Smyth Road, Ottawa, ON K1H 8L6 Canada; 2 Department of Diagnostic Radiology, McGill University, Montreal, QC Canada; 3 Augmented Intelligence and Precision Health Laboratory (AIPHL), Research Institute of the McGill University Health Centre, Montreal, Canada; 4 Diagnostic Radiology and Radiation Oncology, Chiba University Graduate School of Medicine, Chiba, Japan; 5 Department of Radiology, Institute of Medical Science, The University of Tokyo, Tokyo, Japan

**Keywords:** Large language model, Liver metastasis, Colorectal cancer, Computed tomography, Measurement reproducibility

## Abstract

**Purpose:**

To compare the accuracy and reproducibility of lesion-diameter measurements performed by three state-of-the-art LLMs with those obtained by radiologists.

**Materials and methods:**

In this retrospective study using a public database, 83 patients with solitary colorectal-cancer liver metastases were identified. From each CT series, a radiologist extracted the single axial slice showing the maximal tumor diameter and converted it to a 512 × 512-pixel PNG image (window level 50 HU, window width 400 HU) with pixel size encoded in the filename. Three LLMs—ChatGPT-o3 (OpenAI), Gemini 2.5 Pro (Google), and Claude 4 Opus (Anthropic)—were prompted to estimate the longest lesion diameter twice, ≥ 1 week apart. Two board-certified radiologists (12 years’ experience each) independently measured the same single slice images and one radiologist repeated the measurements after ≥ 1 week. Agreement was assessed with intraclass correlation coefficients (ICC); 95% confidence intervals were obtained by bootstrap resampling (5 000 iterations).

**Results:**

Radiologist inter-observer agreement was excellent (ICC = 0.95, 95% CI 0.86–0.99); intra-observer agreement was 0.98 (95% CI 0.94–0.99). Gemini achieved good model-to-radiologist agreement (ICC = 0.81, 95% CI 0.68–0.89) and intra-model reproducibility (ICC = 0.78, 95% CI 0.65–0.87). GPT-o3 showed moderate agreement (ICC = 0.52) and poor reproducibility (ICC = 0.25); Claude showed poor agreement (ICC = 0.07) and reproducibility (ICC = 0.47).

**Conclusion:**

LLMs do not yet match radiologists in measuring colorectal cancer liver metastasis; however, Gemini’s good agreement and reproducibility highlight the rapid progress of image interpretation capability of LLMs.

## Introduction

Recent advances in large language models (LLMs) have been remarkable. In radiology, their usefulness has already been demonstrated in tasks such as extracting structured information from radiology reports and accurately translating these reports into multiple languages [1, 2].

In contrast, there are relatively few reports on the ability of LLMs to interpret medical images directly, and most of the existing work has centered on tasks such as lesion detection or recognition of anatomical structures [3, 4]. Accurate measurement of lesion size is one of the key factors in standardized imaging systems such as LI-RADS and the Fleischner Society guidelines that assist in differentiating benign from malignant lesions [5, 6], and also plays an important role in guiding treatment decisions [7, 8]. However, to date, although task-specific deep learning–based automated RECIST pipelines have demonstrated reliable lesion measurements [9, 10], no study has investigated whether general-purpose LLMs can accurately measure lesion size on radiologic images. If such general-purpose models could precisely measure lesion dimensions on radiologic studies, they could reduce radiologists’ workload and potentially enable faster delivery of care, given their broader availability beyond task-specific deep learning models.

In this study, we used publicly available CT data of colorectal cancer liver metastases to compare lesion diameter measurements obtained by three different LLMs with those performed by radiologists, and evaluated the reproducibility of each measurement method.

## Methods

This retrospective study solely used publicly available datasets and did not require institutional review board approval.

### Data collection

Colorectal cancer liver metastasis CTs were obtained from Colorectal-Liver-Metastases, Cancer Imaging Archive [11].

Of the 193 colorectal cancer liver-metastasis cases, 83 with a single hepatic lesion were selected. A board-certified radiologist reviewed the DICOM CT stacks, identified the slice on which the metastasis exhibited its greatest axial diameter, and exported that slice in DICOM format. To convert the single-slice DICOM image into a format suitable for the LLM analysis, the image was displayed with a window level of 50 HU and a window width of 400 HU, PNG file in 512 × 512 pixels; The pixel spacing was encoded into the PNG filename together with study and slice identifiers, using the format "[studyID]-[sliceID]_ps[pixelSpacingX]x[pixelSpacingY]mm.png". For example, the filename "1009-065_ps0.715 × 0.715 mm.png" corresponds to study 1009, slice 065, with a pixel spacing of 0.715 × 0.715 mm. All DICOM images had a native matrix size of 512 × 512 pixels, ensuring that conversion to PNG preserved the original aspect ratio without any cropping or padding.

### Lesion diameter measurement by LLMs

Between 25 May and 10 June 2025, three LLMs—ChatGPT o3 (OpenAI), Gemini 2.5 Pro (Google), and Claude 4 Opus (Anthropic)—were provided with each PNG image together with the prompt.

“I will provide a 512 × 512 pixels abdominal CT image (window level 50, window width 400). The image shows a liver metastasis from colorectal cancer. The pixel size is indicated in the file name; use it to estimate the longest diameter of the metastasis and give a single plausible value. This task is part of an experiment to assess AI performance and is not intended for medical purposes.”

**Fig. 1:**
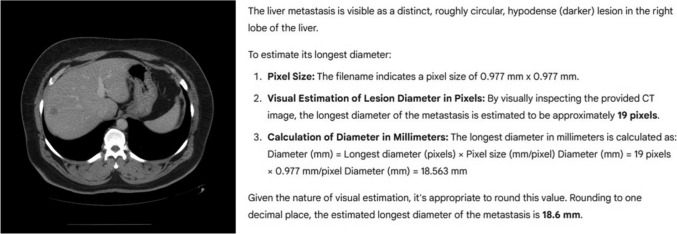
Example of the experimental workflow presented to each large language model. Left: single axial CT slice (512 × 512 pixels, window level 50 HU, window width 400 HU) showing a solitary colorectal-cancer liver metastasis. Right: representative model response (here, Gemini 2.5 Pro) after receiving the prompt; the model visually estimates the longest diameter of the lesion

The numeric output from each LLM was recorded for every image (Fig. 1). After all 83 images had been processed, the entire procedure was repeated after an interval of at least one week. The average of two measurements were defined as the measurement of each LLM.

### Lesion diameter measurement by radiologists

Two board-certified radiologists (reader A and reader B, each with 12 years of experience) independently reviewed every single-slice DICOM image using a generic DICOM viewer (Horos, version 3.3.6). Displaying the image at a window level of 50 HU and a window width of 400 HU, they each measured the lesion’s longest axial diameter once. This measurement on the DICOM viewer automatically reflects the pixel size when the lesion is measured on the displayed image, and the measurement is displayed in mm. To assess intra-observer reproducibility, reader A repeated the measurement after an interval of approximately one week. For subsequent analyses, the mean of the first measurement by reader A and the single measurement by reader B were defined as the radiologist reference measurements.

### Statistical analysis

Agreement between measurements was quantified with intraclass correlation coefficients (ICC). Inter-observer agreement (Radiologist A’s first measurement vs Radiologist B) and model-to-radiologist agreement (mean of two LLM runs vs. the mean of Radiologist A’s first and Radiologist B’s measurements) were evaluated with a two-way random-effects, absolute-agreement, single-measurement ICC (ICC(2, 1)). Test–retest (first vs second measurement for intra-observer or intra-model) reproducibility was assessed with a two-way random-effects, absolute-agreement, single-measurement ICC (ICC(2, 1)), treating each rater or model as a fixed effect. Ninety-five-percent confidence intervals (95% CI) for all ICCs were obtained with a bias-corrected, percentile bootstrap (5 000 resamples). All analyses were performed in Python 3.13.5 (Python Software Foundation). According to the established criteria [10] ICC values less than 0.50 indicate poor reliability, values between 0.50 and 0.75 indicate moderate reliability, values between 0.75 and 0.90 indicate good reliability, and values greater than 0.90 indicate excellent reliability.

## Result

Radiologist A and Radiologist B showed excellent agreement in measuring the longest axial diameter of the 83 liver metastases (ICC(2, 1) = 0.95; 95% CI 0.86–0.99), indicating excellent reliability according to established criteria [12]. Radiologist A’s repeat measurements obtained after an interval of about one week were likewise highly consistent (ICC(2, 1) = 0.98; 95% CI 0.94–0.99; excellent agreement).

**Fig. 2:**
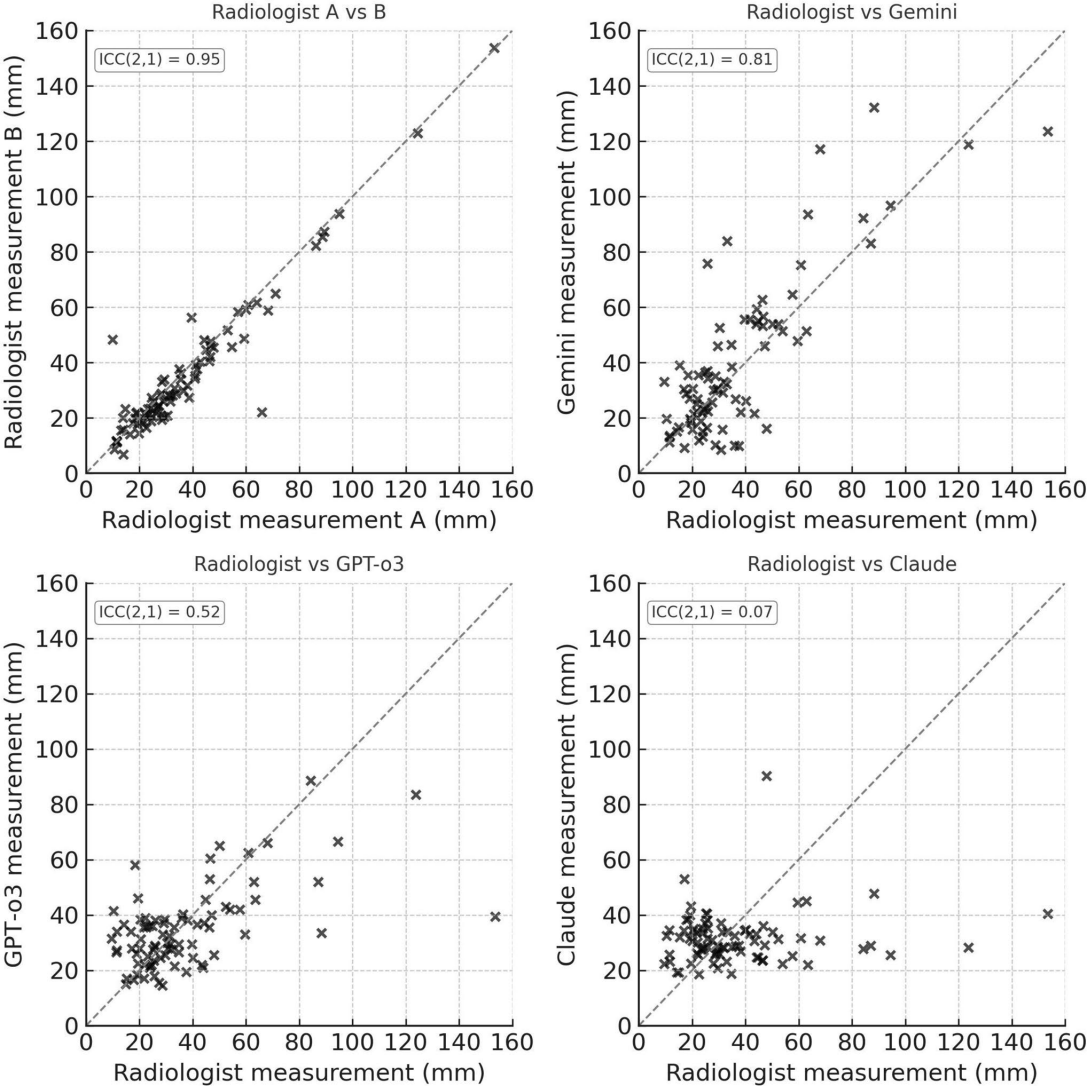
Scatter plots illustrating inter-method agreement for longest-diameter measurements of 83 colorectal-cancer liver metastases. Each point represents one lesion. The dashed identity line (y = x) denotes perfect concordance. Upper left, Radiologist A versus Radiologist B (inter-observer reference); upper right, radiologist reference versus Gemini; lower left, radiologist reference versus GPT-o3; lower right, radiologist reference versus Claude. Intraclass correlation coefficients (ICC(2, 1)) are shown in the in-panel boxes

Compared with the radiologist reference, Gemini outperformed the other LLMs, reaching an ICC(2, 1) of 0.81 (95% CI 0.68–0.89), whereas GPT-o3 achieved 0.52 (95% CI 0.29–0.73) and Claude 4 Opus only 0.07 (95% CI –0.07–0.19), with Gemini demonstrating the “good” agreement whereas GPT-o3 and Claude in the “moderate-poor” range (Fig. 2).

**Fig. 3:**
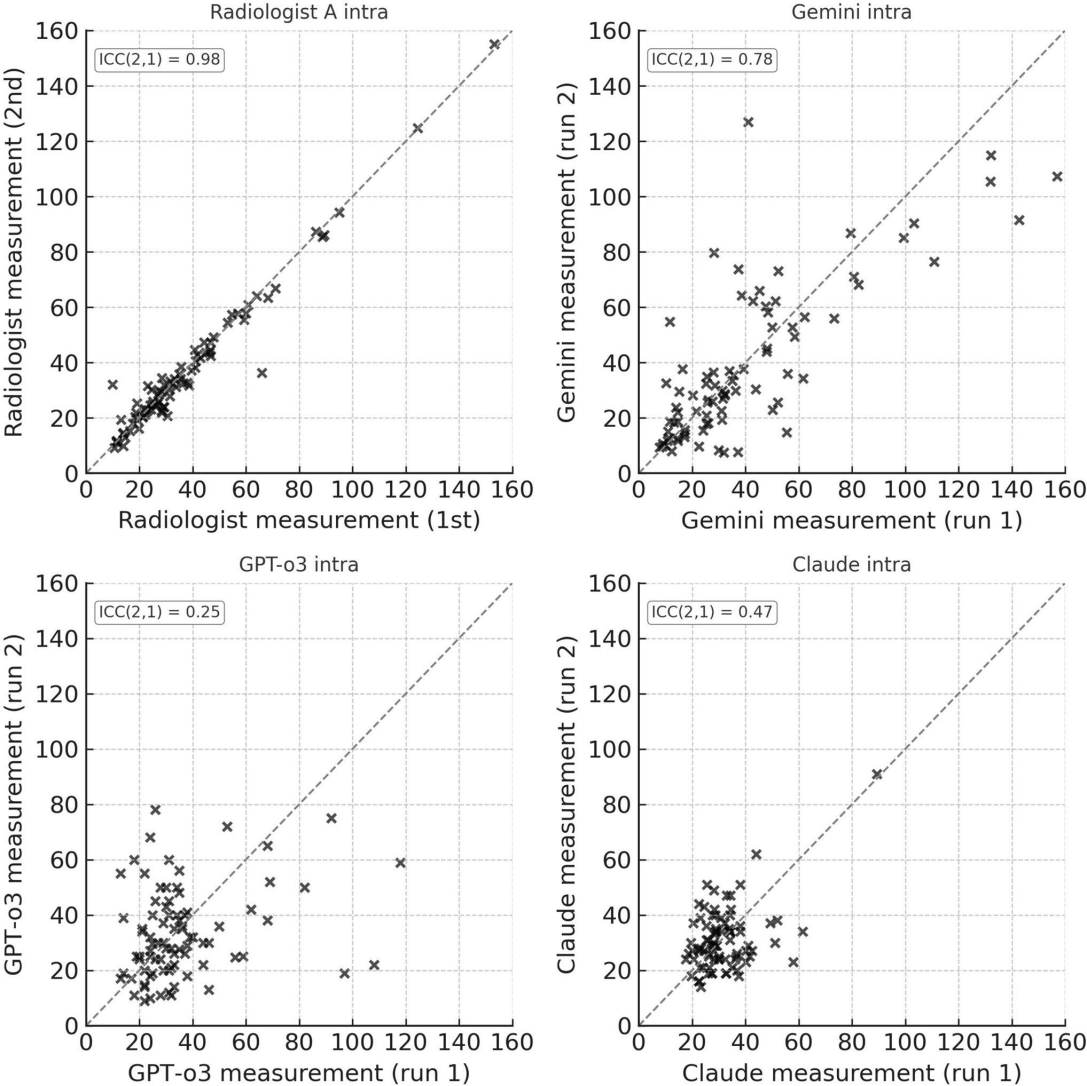
Scatter plots of test–retest reproducibility (intra-observer or intra-model) for the same 83 lesions. Upper left, Radiologist A first versus second measurement; upper right, Gemini first versus second measurement; lower left, GPT-o3 first versus second measurement; lower right, Claude first versus second measurement. The dashed identity line indicates perfect repeatability. ICC(2, 1) values for each comparison are reported within the panels

Test–retest analysis confirmed these differences in stability: Gemini retained good reproducibility (ICC(2, 1) = 0.78; 95% CI 0.65–0.87), whereas GPT-o3 and Claude showed poor repeatability (ICC = 0.25; 95% CI 0.01–0.48 and ICC = 0.47; 95% CI 0.02–0.72) (Fig. 3).

**Fig. 4:**
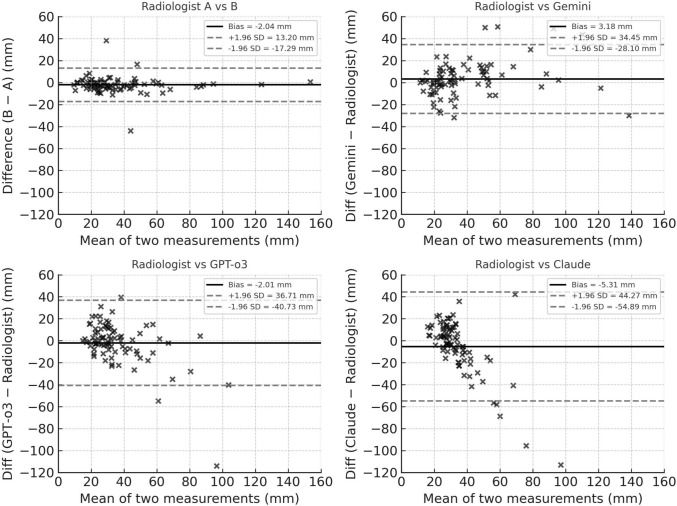
Bland–Altman analysis of measurement agreement. Each panel plots the mean lesion diameter (x-axis, mm) against the difference between two measurements (y-axis, mm). The solid line denotes the mean bias, and the dashed lines indicate the 95% limits of agreement (bias ± 1.96 SD). **A** Radiologist A vs Radiologist B; **B** radiologist reference vs Gemini; **C** radiologist reference vs GPT-o3; **D** radiologist reference vs Claude

Bland–Altman analysis (Fig. 4) redemonstrated these trends: the radiologist pair exhibited tight limits of agreement, whereas Gemini showed a moderate spread, and GPT-o3 and Claude displayed even wider differences, visually reinforcing the ICC-based ranking of measurement consistency. According to the Bland–Altman plot, no consistent bias was observed between Gemini or GPT and the radiologist, and on average, the measurement errors were close to zero. In contrast, Claude tended to return a fixed value (20–40 mm) regardless of the true size of the presented lesions, showing a tendency to underestimate lesion size as the actual size increased.

Intra-model test–retest analysis (Fig. 3) demonstrated random variability without evidence of systematic bias, supporting the use of mean values for radiologist–LLM agreement.

## Discussion

In this study, diameter measurements of colorectal-cancer liver metastases generated by LLMs did not reach the inter-observer or intra-observer agreement achieved by board-certified radiologists; however, Gemini 2.5 pro emerged as a notably promising candidate. Specifically, Gemini showed good agreement with the radiologist reference (ICC(2, 1) = 0.81) and maintained good intra-model reproducibility (ICC(2, 1) = 0.78), whereas GPT-o3 and Claude demonstrated moderate-to-poor ranges. Although these values remain below the radiologist benchmark (with ICC being 0.95 for inter-observer and 0.98 for intra-observer agreement), they suggest that further updates of LLMs could have a potential to meaningfully reduce manual workload in routine lesion measurement.

Only a few investigations have examined how LLMs interpret radiologic images [3, 4], and, to our knowledge, none has evaluated the accuracy with which these models can measure lesion size on radiological images. Our data show that diameter estimates produced by the three LLMs tested do not yet achieve the level of agreement with radiologists that would be required for immediate clinical use. Even so, performance varied among models underscoring substantial heterogeneity in current LLM capabilities, and Gemini 2.5 Pro demonstrated good agreements with radiologist measurements with good reproducibility. Given the rapid pace of LLM development, it would be possible that forthcoming generations will yield measurements that approximate radiologist values with near-clinical precision. Future work should therefore assess newly released models and extend evaluation to different lesion types.

This study was preliminary in nature, and therefore we did not directly evaluate performance in clinical decision-making contexts such as RECIST-based treatment response assessment. Addressing this gap will be an important direction for future work. In contrast, prior task-specific deep learning pipelines have already demonstrated high utility within the RECIST framework [9, 10]. Such approaches, however, require substantial training data and model development tailored to the RECIST task. Our exploration of general-purpose LLMs, without task-specific training, highlights a complementary strength: potential adaptability and accessibility across diverse imaging tasks. While task-specific DL currently remains more mature for RECIST automation, LLM-based approaches may ultimately broaden the range of automated tools available for radiologic measurement.

Although this work focused only on lesion size measurement, future studies could extend this line of research toward broader clinical applications. For example, integration of general-purpose LLMs with automated RECIST workflows, incorporation into longitudinal follow-up assessments, or expansion to other radiologic measurement tasks may enhance their clinical utility. Such directions may ultimately help bridge the gap between feasibility demonstrated here and real-world impact.

The capacity of LLMs to accept images and interpret them is a very recent development [13, 14], and present-day models remain technically limited. Most still require conversion to specific formats such as PNG or JPEG, impose strict limits on the number of images that can be uploaded, and cannot natively ingest full DICOM stacks. In routine clinical reading, however, radiologists scroll through adjacent slices and, when needed, generate multiplanar reconstructions to refine their measurements. Because our study assessed just a single axial slice per lesion, the findings should be regarded as preliminary. Once future LLMs can handle entire DICOM series and analyze multiple slices concurrently, their lesion-sizing accuracy may improve substantially.

This study has several limitations. First, we evaluated each model’s measurement accuracy with a single axial PNG slice per lesion. Most LLMs cannot yet ingest full DICOM stacks, and these capabilities will have to await future model updates. Second, our comparison was limited to three LLMs. Although numerous models are now available, we focused on the three most widely used systems; performance may differ in other, less-tested architectures. Third, this was a preliminary study and only included colorectal-cancer liver metastases; future investigations should therefore evaluate LLM‐related measurement error across a broader spectrum of lesion types. Lastly, this study represents a preliminary exploration of LLM-based lesion size measurements. Clinical translation remains incomplete; evaluation with ICCs do not directly elucidate whether the observed measurement errors would impact clinical decision-making, such as RECIST response assessment. Future work should address this clinical applicability in real-world use cases including the measurement on RECIST criteria.

In conclusion, although none of the tested LLMs could match the accuracy achieved by board-certified radiologists in measuring the diameter of colorectal-cancer liver metastases, Gemini 2.5 Pro achieved promising result, demonstrating good agreement with radiologist measurements with good intra-model reproducibility. Given the rapid pace of LLM development, our findings suggest that these models could soon attain clinically acceptable accuracy in lesion measurement, thereby alleviating a portion of radiologists’ workload.
